# Editorial: The application of complex training in sports: theory, practice, and impact

**DOI:** 10.3389/fspor.2026.1875978

**Published:** 2026-06-02

**Authors:** Limingfei Zhou, Kaixiang Zhou, Junhong Zhou

**Affiliations:** 1 School of Strength and Conditioning Training, Beijing Sport University, Beijing, China; 2 College of Physical Education and Health Science, Chongqing Normal University, Chongqing, China; 3 Hinda and Arthur Marcus Institute for Aging Research, Hebrew SeniorLife, Department of Medicine, Harvard Medical School, Boston, MA, United States

**Keywords:** athletic performance, complex training, fatigue monitoring, post-activation performance enhancement, sport-specific adaptation

Complex training has evolved from a narrowly defined strength-power method into a broader performance framework that links resistance exercise, plyometrics, explosive execution, warm-up design, fatigue management, and sport-specific adaptation. The articles collected in this Research Topic reflect that evolution. Across basketball, swimming, badminton, martial arts, volleyball, distance running, soccer, triathlon, and university health settings, the contributions move the field beyond the simple question of whether complex training “works”. Instead, they address how it should be defined, when it is most effective, how it can be implemented across populations and sports, and how its effects should be monitored over time.

A useful starting point is the synthesis of evidence and the clarification of terminology. A systematic review and meta-analysis on basketball players showed that complex training can improve key basketball-relevant outcomes, particularly change-of-direction speed and jump performance, thereby reinforcing its value in intermittent team sports (Kambitta Valappil et al.). At the same time, a commentary on that review reminds us that progress in this area depends not only on positive findings, but also on conceptual precision (Thapa et al.). The commentary highlights the importance of clearer definitions and more careful interpretation, an issue that remains central in a literature where “complex”, “contrast”, and related terms are still sometimes used inconsistently.

Several articles then advance the acute-performance perspective by examining how different training or warm-up configurations influence readiness and immediate output. In elite swimmers, complex training produced the most favorable post-activation performance enhancement profile, while also demonstrating that heavy resistance, plyometric, and complex modes may each have distinct optimal recovery windows (Ma et al.). This finding is relevant for practitioners, because it shifts attention from the intervention alone to the interaction between intervention and timing. In children soccer players, preparatory activity structure also matters, with different warm-up approaches influencing agility, sprint-related outcomes, and kicking performance in different ways (Hernandez-Martínez et al.). Likewise, a modified field test in non-elite badminton players contributes an ecologically grounded approach to fatigue induction and monitoring, reminding us that performance enhancement must always be interpreted alongside the fatigue it may produce (Huang et al.).

This Research Topic also highlights chronic, sport-specific adaptations. In elite martial arts athletes, a sequenced heavy-to-explosive stimulus improved multiple indicators of lower-body strength and power, supporting the usefulness of high-low load integration in technically demanding sports (Chen et al.). In adolescent female badminton players, complex training outperformed traditional resistance training for explosive and multidirectional performance, indicating its relevance in youth female athletes (Hu et al.). Similarly, work in adolescent distance runners extends the value of complex training into endurance sport, showing that neuromuscular-oriented methods may contribute not only to power expression but also to running economy (Yu et al.). In elite male volleyball players, the comparison of unilateral and bilateral formats adds a programming dimension, suggesting that the structural organization of complex training may shape transfer to short sprinting and multidirectional performance (Wang et al.).

This Research Topic also shows that the practical ecosystem around complex training includes adjacent modalities rather than complex training alone. In male collegiate badminton players, sand-based plyometric training demonstrates that training surface and context can modify how explosive training is delivered without necessarily changing its overall effectiveness (Deng et al.). A multilevel meta-analysis of traditional Chinese mind-body training in university students broadens the conversation further, reminding us that training design should also be population-sensitive and outcome-specific, especially when the target is general physical health rather than elite performance alone (Cui et al.).

Finally, this Topic also points toward longitudinal monitoring and individualized interpretation. A further accepted contribution in elite triathletes suggests that training adaptation cannot be understood solely through prescribed load or isolated outcome measures. By linking subjective and objective load indicators with biomarker responses and performance changes, this study highlights a future direction for the field: complex training and related performance methods should be embedded within individualized monitoring systems that account for inter-individual variability, recovery status, and the possibility that the same external stimulus may lead to very different internal responses.

Taken together, the articles in this Research Topic suggest that the field is continuing to develop in a more integrated way. The central issue is no longer whether combining high-force and high-velocity stimuli can enhance performance; the accumulating evidence indicates that it can. Questions that now warrant further attention concern definition, sequencing, dose, timing, sport specificity, developmental stage, and monitoring. Future studies should continue to standardize terminology, compare different complex-training configurations, examine longer intervention periods, and integrate performance outcomes with internal-load, fatigue, and recovery markers. In doing so, researchers and practitioners may be better positioned to translate complex training from an effective method into an individualized and context-responsive component of sport preparation.

